# Magnetic-field-induced robust zero Hall plateau state in MnBi_2_Te_4_ Chern insulator

**DOI:** 10.1038/s41467-021-25002-x

**Published:** 2021-07-30

**Authors:** Chang Liu, Yongchao Wang, Ming Yang, Jiahao Mao, Hao Li, Yaoxin Li, Jiaheng Li, Haipeng Zhu, Junfeng Wang, Liang Li, Yang Wu, Yong Xu, Jinsong Zhang, Yayu Wang

**Affiliations:** 1 State Key Laboratory of Low Dimensional Quantum Physics, Department of Physics, Tsinghua University, Beijing, China; 2 Beijing Academy of Quantum Information Sciences, Beijing, China; 3 Beijing Innovation Center for Future Chips, Tsinghua University, Beijing, China; 4 Wuhan National High Magnetic Field Center and School of Physics, Huazhong University of Science and Technology, Wuhan, China; 5 School of Materials Science and Engineering, Tsinghua University, Beijing, China; 6 Tsinghua-Foxconn Nanotechnology Research Center, Department of Physics, Tsinghua University, Beijing, China; 7 Department of Mechanical Engineering, Tsinghua University, Beijing, China; 8 RIKEN Center for Emergent Matter Science, Wako, Saitama, Japan; 9 Frontier Science Center for Quantum Information, Beijing, China

**Keywords:** Electronic properties and materials, Topological insulators

## Abstract

The intrinsic antiferromagnetic topological insulator MnBi_2_Te_4_ provides an ideal platform for exploring exotic topological quantum phenomena. Recently, the Chern insulator and axion insulator phases have been realized in few-layer MnBi_2_Te_4_ devices at low magnetic field regime. However, the fate of MnBi_2_Te_4_ in high magnetic field has never been explored in experiment. In this work, we report transport studies of exfoliated MnBi_2_Te_4_ flakes in pulsed magnetic fields up to 61.5 T. In the high-field limit, the Chern insulator phase with Chern number *C* = −1 evolves into a robust zero Hall resistance plateau state. Nonlocal transport measurements and theoretical calculations demonstrate that the charge transport in the zero Hall plateau state is conducted by two counter-propagating edge states that arise from the combined effects of Landau levels and large Zeeman effect in strong magnetic fields. Our result demonstrates the intricate interplay among intrinsic magnetic order, external magnetic field, and nontrivial band topology in MnBi_2_Te_4_.

## Abstract (web summary)

The antiferromagnetic topological insulator MnBi_2_Te_4_ exhibits Chern and axion insulator phases at low magnetic field; however, its behaviour in high magnetic field has remained unexplored. Here, using transport measurements at high magnetic field, the authors report a zero Hall plateau composed of two counter-propagating edge channels.

## Introduction

A remarkable breakthrough in the field of topological quantum matter is the discovery of topological insulators (TIs) with nontrivial bulk band topology and metallic boundary states^1–8^. For two-dimensional (2D) TI with time-reversal symmetry (TRS), the helical edge states give rise to the quantum spin Hall (QSH) effect^3,5,9–11^. When TRS is broken in magnetic TI, the quantum anomalous Hall (QAH) effect with chiral edge state emerges^6–8,12–14^. Because the QAH effect originates from the topological Chern band rather than Landau levels^13,14^, it is now called the Chern insulator phase to distinguish it from conventional quantum Hall (QH) insulator^2,15,16^. Previous efforts on the Chern insulator mainly focused on its realization in zero magnetic field^6–8,12^. An intriguing question that has yet to be addressed experimentally is the fate of the Chern insulator in ultra-high magnetic fields^17,18^. It is likely that the QAH plateau will not survive forever because extreme conditions may induce other topological phases, as exemplified by the fractional QH effect^19^. Recently, it was demonstrated that helical phases analogous to the QSH phase exist in charge-neutral graphene^20,21^ and non-symmorphic KHgSb crystal^22^ in strong magnetic fields, which are characterized by quantized longitudinal resistance (*R*_*xx*_) and zero Hall resistance (*R*_*yx*_) plateau in certain magnetic fields and gate voltage (*V*_*g*_) ranges. Inspired by these discoveries, it is imperative to find out the fate of the Chern insulator phase in MnBi_2_Te_4_ in strong magnetic fields, as has been discussed theoretically for magnetic TIs^23,24^.

**Fig. 1:**
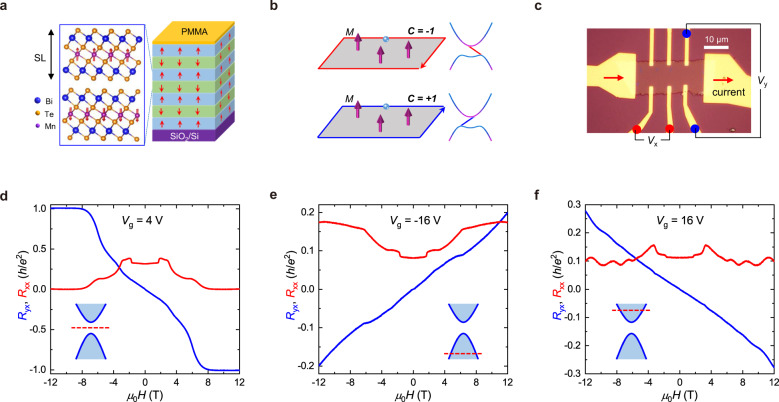
Basic properties of a 7-SL MnBi_2_Te_4_. **a** Schematic crystal and magnetic structures of the 7-SL MnBi_2_Te_4_ device. **b** Configurations of chiral edge state in the Chern insulator with Chern number *C* = −1 and +1. The opposite chirality of the edge state is marked by red and blue lines with arrows. The magenta arrows denote the magnetic moments. The schematic electronic structures for the two cases are shown on the right, with the opposite slope of the linear band representing opposite chirality. **c** Optical image of Device #7-SL-1 and the measurement setup. **d** Magnetic field dependent *R*_*xx*_ (red) and *R*_*yx*_ (blue) at *V*_*g*_ = 4 V and *T* = 2 K. The Chern insulator phase is realized when the magnetic field is above 8 T, which is characterized by the *R*_*yx*_ = −*h*/*e*^2^ plateau and *R*_*xx*_ = 0. **e** As *V*_g_ is tuned to −16 V, the transport is dominated by hole-type carriers. The jumps in *R*_*xx*_ at a magnetic field of around 1.8 T, 4 T, and 7 T correspond to the successive flips of Mn^2+^ moments in different SLs. **f** At *V*_*g*_ = 16 V, the *E*_*F*_ is tuned to the conduction band and the transport exhibits characteristic features of the 2D electron gas. The insets in **d** to **f** roughly show the position of *E*_*F*_ at according *V*_*g*_.

The recently discovered MnBi_2_Te_4_ combines intrinsic magnetism and nontrivial topology in one material^25–37^, providing an ideal platform for exploring the topological phenomenon in extreme physical conditions. Figure 1a displays the schematic magnetic and crystal structure of MnBi_2_Te_4_, where the Mn^2+^ magnetic moments have ferromagnetic (FM) alignments within each septuple layer (SL) and antiferromagnetic (AFM) coupling between neighboring SLs. In the 2D limit, it exhibits the QAH insulator^30^ and axion insulator^29^ phases for odd-number and even-number of SLs. When FM order is induced by magnetic fields, the parity-time (PT) symmetry is broken and the bulk of MnBi_2_Te_4_ becomes a Weyl semimetal^26,34,38^. In thin MnBi_2_Te_4_ flakes, the bulk Weyl semimetal band develops 2D quantum well states due to the quantum confinement along the *c*-axis. The topologically nontrivial quantum well subbands lead to a robust Chern insulator state with a quantized Hall plateau that persists to relatively high temperatures for magnetic field *μ*_0_*H* > 8 T^29–31^. A unique feature of the Chern insulator phase in MnBi_2_Te_4_ lies in the negative sign of the Chern number (*C* = −1) in positive magnetization, which is distinguishable from the *C* = +1 Chern insulator phase in Cr-doped or V-doped TIs^6–8,12^. Figure 1b illustrates the spatial configurations of the chiral edge state and the band structures for the two cases. Phenomenologically, the opposite sign of the Chern number arises from the opposite effective magnetic field due to the interplay between magnetic order and spin-orbit coupling^39,40^. Because magnetic field couples with both magnetic moment and electron spin, for certain spin configurations of band structure, the combination of Landau levels and a sufficiently strong Zeeman energy may lead to topological quantum phenomena such as TRS-broken QSH effect or the quantum pseudospin Hall effect^23,24^ which have been proposed theoretically but never been realized in the experiment.

In this work, we report transport studies on exfoliated MnBi_2_Te_4_ in pulsed magnetic fields up to 61.5 T. Unexpectedly, the *C* = −1 phase evolves into a zero Hall plateau state characterized by a broad *R*_*yx*_ = 0 plateau and insulating *R*_*xx*_ in an ultrahigh magnetic field. Nonlocal transport measurements and theoretical calculations demonstrate the transport of this zero Hall plateau state is composed of two counter-propagating edge states with opposite Chern numbers, which arise from the FM order and the joint roles of Landau levels and Zeeman effect. The robust zero Hall plateau state represents a topological phenomenon that is unavailable in 2D electron-gas or hole-gas with conventional QH effect.

## Results

The MnBi_2_Te_4_ devices studied in this work are mechanically exfoliated few-layer flakes fabricated into field-effect transistor devices on SiO_2_/Si substrates that act as the bottom gate. The details of fabrication and transport measurements in pulsed magnetic fields are described in the method session. All the data in the main text are collected on a 7-SL device (#7-SL-1), and its photo is displayed in Fig. 1c. We first characterize the low-field transport properties at temperature *T* = 2 K in varied *V*_*g*_s, and three representative curves are shown in Fig. 1d to 1f (see Supplementary Fig. 1 for the full data set). The insets schematically illustrate the Fermi level (*E*_*F*_) position for each *V*_*g*_. The most pronounced feature is that for *V*_*g*_ = 4 V when *E*_*F*_ is in the Chern bandgap at FM state. At the low magnetic field side, *R*_*xx*_ exhibits three jumps due to the successive flips of Mn^2+^ moments, which is consistent with previous reports on few-layer MnBi_2_Te_4_ devices^29–31^. Meanwhile, |*R*_*yx*_| progressively grows with the magnetic field, and the slope is mainly determined by the magnetization. A well-defined quantum plateau forms at −*h*/*e*^2^ in *R*_*yx*_ for *μ*_0_*H* > 8 T, accompanied by the rapid decrease of *R*_*xx*_ to zero. These are consistent with the Chern insulator behaviors in previously reports^29–31^. As *V*_*g*_ is tuned away from 4 V to either side, *E*_*F*_ moves out of the bandgap, and carriers from the 2D subbands appear. The quantization of the Chern insulator phase is suppressed, and the negative (positive) slopes of the Hall traces at *V*_*g*_ = +16 V (−16 V) indicate the existence of electron (hole) type carriers. According to the linear slope, the mobility in the electron-type and the hole-type regime is estimated to be 3114 and 2098 cm^2^/Vs, respectively.

**Fig. 2:**
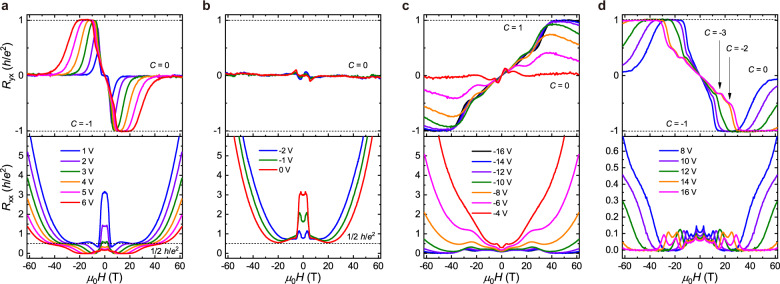
Transport properties in pulsed magnetic field up to 61.5 T. **a** Magnetic field dependent *R*_*xx*_ and *R*_*yx*_ at 1 V ≤ *V*_*g*_ ≤ 6 V. At *V*_*g*_ = 4 V, the *C* = −1 state is completely suppressed for *μ*_0_*H* > 30 T, followed by the *C* = 0 state characterized by a broad zero Hall plateau. The black dashed line denotes the *R*_*xx*_ = 0.5 *h*/*e*^2^ plateau. **b** Transport properties at −2 V ≤ *V*_*g*_ ≤ 0 V. Zero Hall plateau exist in a broad magnetic field range over 50 T. **c** Transport behaviors in the 2D hole gas regime. With the decrease of *V*_*g*_, QH plateaus with a positive Chern number start to form. At *V*_*g*_ = −16 V, *R*_*yx*_ forms a wide Hall plateau at *h*/*e*^2^ and *R*_*xx*_ drops to zero. **d** Characteristic transport behaviors of the 2D electron gas. With the increase of *V*_*g*_ from 8 V to 16 V, the *C* = −1 Hall plateau onsets at a higher magnetic field, becomes broader and approaches the *C* = 0 plateau only at the high-field limit. Electron-type QH plateaus with higher Chern numbers *C* = −2 and −3 also start to form.

When we extend the transport measurements to much higher magnetic fields, some totally unexpected features start to emerge. According to the characteristic behavior of *R*_*yx*_, the entire *V*_g_ range can be divided into four different regimes. As shown in Fig. 2a, the most remarkable feature of the high-field data is that the *C* = −1 state only survives in a range of about 10 T. With further increase of magnetic fields, *R*_*yx*_ drops rapidly from the −*h*/*e*^2^ plateau and, even more surprisingly, a very broad *R*_*yx*_ = 0 plateau forms in the high-field regime. Meanwhile, *R*_*xx*_ takes off from the zero value, develops a shoulder ~0.5 *h*/*e*^2^ at the onset field of the zero plateau, and then increases again in higher magnetic fields. Remarkably, all the *R*_*xx*_ curves for varied *V*_g_s tend to converge to the 0.5 *h*/*e*^2^ value (marked by the broken line) near the onset magnetic field of the zero Hall plateau. With the decrease of *V*_*g*_, the high field zero Hall plateau is universally present and becomes even broader. As shown in Fig. 2b for *V*_*g*_ from −2 V and 0 V, the zero Hall plateau spans an incredibly wide field range from 10 T to the highest available field of 61.5 T. In Supplementary Fig. 2, we display the magnetic field-dependent *R*_*xx*_ and *R*_*yx*_ at *V*_*g*_ = 4 V for varied temperatures, in which a clear tendency of *R*_*xx*_ saturation at 0.5 *h*/*e*^2^ is observed at low temperatures. Both the *C* = −1 phase and the zero Hall plateau state exhibit high robustness against thermal activation, even up to *T* = 20 K. Similar transport behaviors are also observed in other samples with different sizes and thicknesses (#7-SL-2 and #6-SL-1), as shown the Supplementary Fig. 3 to Fig. 7.

When *V*_*g*_ is decreased to more negative values, as shown in Fig. 2c, the Hall traces evolve to that characteristic of a 2D hole gas with an overall positive profile and growing amplitude from *V*_*g*_ = −4 V to −16 V. Meanwhile, *R*_*xx*_ reduces systematically as more holes are injected into the MnBi_2_Te_4_ flake. At *V*_*g*_ = −16 V, *R*_*yx*_ forms a well-defined Hall plateau at *h*/*e*^2^ (*C* = +1) for *μ*_0_*H* > 40 T, whereas *R*_*xx*_ drops to zero within experimental uncertainty. When *V*_*g*_ is tuned to the opposite side with *V*_*g*_ from 8 V to 16 V (Fig. 2d), quantized *R*_*yx*_ plateaus with Chern numbers *C* = −3, −2, and −1 show up, along with apparent quantum oscillations. Remarkably, a unique feature here is that all the plateaus exhibit a strong tendency towards the zero Hall plateau state in the highest magnetic field, regardless of the position of *E*_*F*_, which is quite unusual in conventional QH effect^16^.

**Fig. 3:**
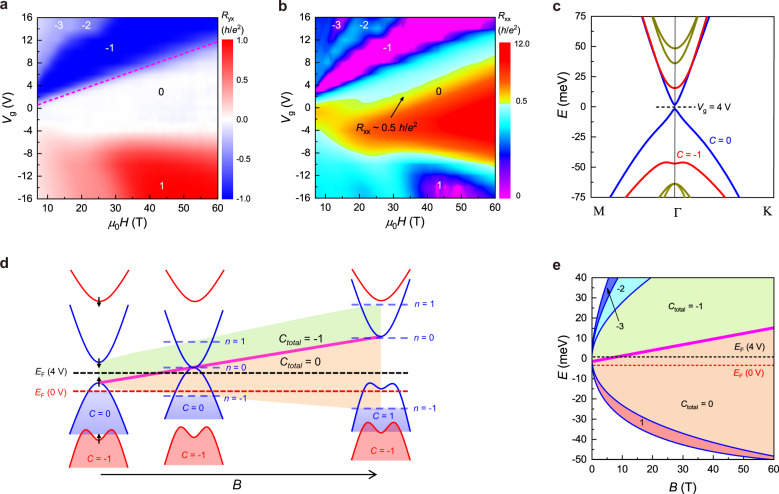
Contour plots of experimental data and theoretical analysis of the *C* = −1 to *C* = 0 phase transition. **a**, **b** Experimental phase diagrams of *R*_*yx*_ and *R*_*xx*_ in the magnetic field and *V*_*g*_ plane. The *C* = 0 phase is the most stable ground state in a strong magnetic field. The magenta broken line denotes the boundary between the *C* = −1 and *C* = 0 phase. The black arrow represents the regime of *R*_*xx*_ ~ 0.5 *h*/*e*^2^ for the helical Chern insulator phase. **c** Calculated band structure of 7-SL MnBi_2_Te_4_ along the M-Γ-K direction when the system is driven into the FM state. The red and blue lines denote the ferromagnetic-order induced Chern band (*C* = −1) and topologically trivial band (*C* = 0), respectively. **d** Schematic illustrations of the edge state formation and the band structure evolution in a magnetic field with Zeeman-effect-induced band inversion. The black and red dashed lines roughly mark the *E*_*F*_ position for *V*_*g* _= 4 V and 2 V. The *C* = −1 to *C* = 0 phase transition occurs once band inversion happens. **e** Calculated Landau level spectrums with Zeeman-effect.

Based on the high field data shown above, in Fig. 3a, b we depict the contour maps of *R*_*yx*_ and *R*_*xx*_ in the *μ*_0_*H* and *V*_*g*_ plane. The most prominent feature is that the zero Hall plateau state occupies the largest portion of the phase diagram. The magenta line marks the boundary between the *C* = −1 and zero Hall plateau state. The biggest puzzles revealed by the high field experiments are the nature of the zero Hall plateau state that prevails in the phase diagram and the underlying mechanism for the phase transition. In recent years, the zero Hall plateau states discovered in graphene and TI have attracted intense attentions^22,41–43^ for their nontrivial origins. However, most previous works focused on Hall conductivity (*σ*_*xy*_) rather than Hall resistivity (*ρ*_*yx*_). Because *σ*_*xy*_ = *ρ*_*yx*_/(*ρ*_*xx*_^2 ^+ *ρ*_*yx*_^2^), any kind of insulating state with large longitudinal resistivity *ρ*_*xx*_ can give rise to a *σ*_*xy*_ = 0 plateau. In contrast, the observation of *R*_*yx*_ = 0 plateau is very rare in experiments. Moreover, a zero Hall plateau state evolved from a Chern insulator or QH state is highly unusual.

The zero Hall plateau and the tendency of *R*_*xx*_ towards 0.5 *h*/*e*^2^ at the onset regime are highly reminiscent of the QSH effect, where the transport is conducted by a pair of helical edge states^3^. Differently, the helical transport in the QSH insulator is protected by TRS^3,9,10^, i.e., the absence of both magnetic field and magnetic order. But here, the zero Hall plateau state is observed when both FM order in a strong magnetic field is present. Therefore, the zero Hall plateau state here represents a distinct insulator state that may host counter-propagating edge states transport with tunable scatterings in magnetic fields. To clarify this issue, we first calculate the band structure of 7-SL MnBi_2_Te_4_ when it is the FM state, as shown in Fig. 3c. According to theories, the breaking of PT-symmetry by FM order drives the bulk of MnBi_2_Te_4_ from an AFM TI into a Weyl semimetal^26,34,38^. In the thin film case, the low-energy physics near *E*_*F*_ is described by four quantum well states, in analogy to the four-band model for the QAH effect in magnetic TIs^13^. In these bands, one pair of subbands (red curves) is already inverted due to FM order, which is responsible for the *C* = −1 phase. The blue bands represent two trivial quantum-well states with an energy gap as small as 3 meV.

The most intuitive explanation for the magnetic-field-induced zero Hall plateau state is to consider the coexistence of a hole-type Landau level (*C* = +1) and the FM-order induced Chern band (*C* = −1). However, because Landau level spacing due to cyclotron motion increases in the magnetic field, the *C* = −1 phase for *E*_F_ lying in the bandgap will persist in a strong magnetic field. Apparently, this is inconsistent with the experimental observation of the *C* = −1 to zero Hall plateau transition. Therefore, it is indispensable to consider the band shift under the Zeeman effect. Because a small Zeeman energy insufficient for bandgap closing will not change the *C* = −1 phase in a certain *V*_*g*_ range with *E*_*F*_ located in the bandgap, a sufficiently large Zeeman effect that can cause a band inversion must be included to explain the absence of persistent *C* = −1 regime and the emergent zero Hall plateau state. The detailed illustrations of the band structure evolutions are displayed in Supplementary Fig. 8.

A closer examination of the spin configuration of each band reveals an enticing possibility of the Zeeman-effect induced band inversion, as shown in Fig. 3d. Because of the strong spin-orbit coupling in MnBi_2_Te_4_, the *z* component of spin angular momentum *s*_*z*_ is no longer a good quantum number. Therefore, we label each state at the Γ point by the total angular momentum *J*_*z*_, which is quantized by *C*_3_ rotational symmetry and is mainly contributed by *s*_z_ because of the small orbital angular momentum of *p*_*z*_ orbitals. The black arrows in the band structure denote the *z* component of *J*_*z*_ for each band at the Γ point. An important point is that spin-up refers to positive *J*_*z*_ or equivalently negative spin magnetic moment *M*_z_. Thus, in an external magnetic field, along with the formation of Landau levels, bands with opposite *J*_*z*_ shift towards the opposite directions. When *E*_*F*_ initially lies near the top of the valence band (e.g., *V*_*g*_ = 0 V), the formation of *n* = 0 Landau level gives rise to a *C* = +1 chiral edge state. In combination with the original FM-order induced *C* = −1 band, a zero Hall plateau forms. Meanwhile, the Zeeman effect pushes the *n* = 0 Landau level upwards in a magnetic field, which leads to the *C* = −1 to zero Hall plateau transition for *E*_*F*_ lying the bandgap (e.g., *V*_*g*_ = 4 V). As the magnetic field is increased further, the Zeeman energy can surpass the gap size of the trivial bands, and a band inversion happens at some critical field. Meanwhile, at the gap closing point, the *n* = 0 Landau level crosses from the valence band to the inverted conduction band. Therefore, the total Chern number of the zero plateau state before and after band inversion does not change. The different shadows in Fig. 3d denote the regimes with different Chern numbers. This scenario is further supported by the calculated Landau level spectrums with the Zeeman effect taken into consideration, as highlighted by the upward phase boundary (magenta line) in Fig. 3e. In Supplementary Fig. 10, we also calculate the Landau level spectrum without the Zeeman effect, which displays apparently a qualitative departure from our experimental phase diagram.

**Fig. 4:**
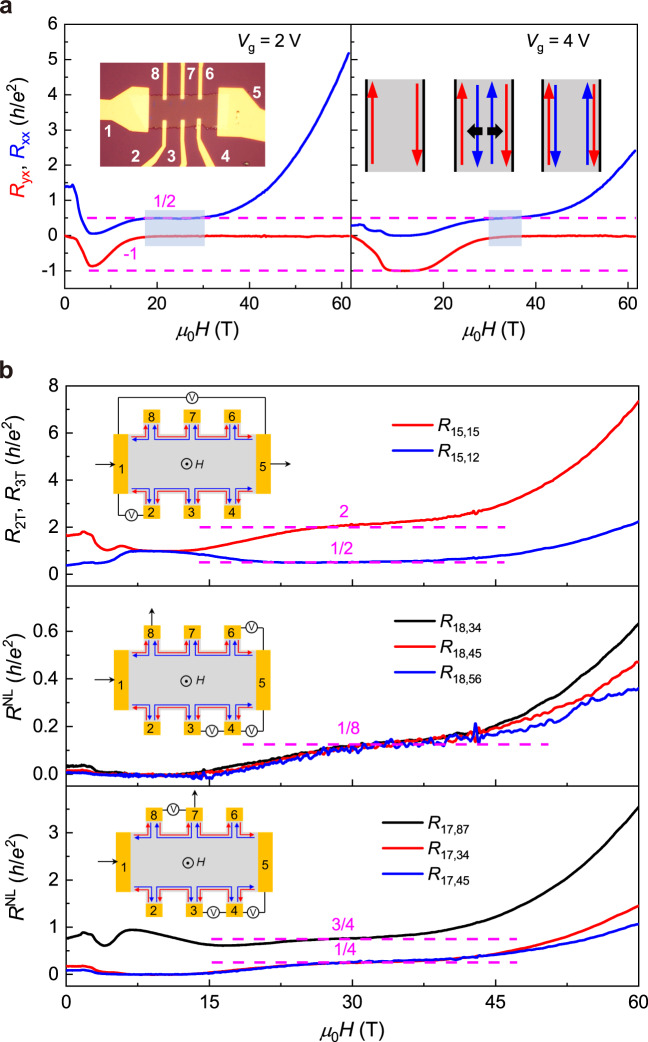
Signatures of helical edge states transport in multiterminal and nonlocal measurements in the *C* = 0 phase. **a** Magnetic field dependent *R*_*xx*_ and *R*_*yx*_ near the *C* = −1 to *C* = 0 phase transition for *V*_*g*_ = 2 V and 4 V. The spatial distribution of edge states in the magnetic field is displayed in the inset. **b** Two-terminal, three-terminal, and nonlocal measurements in various configurations. The inset shows the schematic layout of the experimental setup. The expected values for *R*_2*T*_ and *R*_3*T*_ are 2 *h*/*e*^2^ and 1/2 *h*/*e*^2^, as denoted by the broken lines in the top panels. Middle panel: nonlocal measurements with current flowing through electrodes 1 and 8. The convergence of all three curves near 1/8 *h*/*e*^2^ (denoted by the broken lines) indicates the helical edge transport at the onset of the *C* = 0 phase. Bottom panel: nonlocal measurements in another setup with current flowing through electrodes 1 and 7. Depending on the position of the voltage probes, the resistance values of 1/4 and 3/4 *h*/*e*^2^ are expected, which are confirmed by the experimental results.

For a better comparison with the previously discovered helical QH phase in graphene^20,21^, we zoom in the *R*_*xx*_ and *R*_*yx*_ data in the Chern insulator regime, as displayed in Fig. 4a. At *V*_*g*_ = 2 V, the width of the *R*_*xx*_ ~ 0.5 *h*/*e*^2^ plateau is as broad as 10 T. As *V*_*g*_ is increased to 4 V, the plateau becomes a broad shoulder. In the inset of Fig. 4a, we display the schematic illustrations of measurement setups and the evolution of counter-propagating edge states in a magnetic field. The opposite chirality of the two edge channels at the onset of the zero Hall plateau can naturally explain the zero Hall plateau and the quantized behaviors in *R*_*xx*_. When the new *C* = +1 edge state just appears, its spatial distribution is not confined to the sample boundary^44^. The scattering between the two counter-propagating edge states is weak, thus the *R*_*xx*_ value at the initial stage of the zero Hall plateau state is close to 0.5 *h*/*e*^2^ for scattering-immune helical transport, which is exactly the scenario for stabilizing the QSH edge state with broken TRS^44^. With the further increase of the magnetic field, the edge states are pushed towards the boundaries. Because there is no TRS, the enhanced overlap between the two edge states leads to strong scatterings, giving rise to a rapid increase of *R*_*xx*_. Similar situations of the deviations of *R*_*xx*_ in the high magnetic field are also observed in the helical QH phase in graphene^20,21^.

To further validate the counter-propagating edge state nature of the zero Hall plateau state, we perform multiterminal and nonlocal transport measurements at *V*_*g*_ = 4 V, as displayed in Fig. 4b. The schematic setups of the measurements are shown in the insets. In the situation without scatterings between two edge states, the Landauer-Buttiker formalism renders *R*_2*T*_ = 2 *h*/*e*^2^ and *R*_3*T*_ = 1/2 *h*/*e*^2^. Because each pair of neighboring electrodes contributes a quantum resistance, the final resistance can be calculated by an effective circuit determined by the arrangement of electrodes between source and drain^3,4^. The measured results exactly match such expectations, as marked by the magenta dashed lines. The middle and bottom panels show the results of two different setups for nonlocal measurements, which can directly detect edge state transport^4^. The measured 1/8 *h*/*e*^2^, 3/4 *h*/*e*^2^, and 1/4 *h*/*e*^2^ are the expected values for counter-propagating edge transport with weak scattering. At a high magnetic field where scatterings are enhanced, the zero Hall plateau state evolves towards a trivial insulator when the counter-propagating edge states are fully canceled. However, the nonlocal transport data shows that even up to 61.5 T, there is still a sizeable edge state conduction. For the setup in the middle panel of Fig. 4b, at the onset of the zero Hall plateau when the scattering is weak, the ratio between local and nonlocal resistance is 1:4, which is fully determined by the electrode configurations. The ratio remains at 1:4 at a high magnetic field, which is a characteristic signature of edge state transport. Because a true trivial insulator has no edge conductivity, and residual carriers cannot give rise to such robust zero Hall plateau and do not contribute to any nonlocal signal, the zero Hall plateau state-observed here is a distinct insulator phase from a true trivial insulator. Reproducible non-local data with a much broader plateau width can be seen in another 7-SL sample (#7-SL-3) shown in Supplementary Fig. 11.

## Discussion

The physical picture of emergent Chern band gaps induced by Landau levels and the Zeeman effect gives a comprehensive understanding of the zero Hall plateau state-observed here in thin flakes of MnBi_2_Te_4_ in strong magnetic fields. The key factor is to create a Chern bandgap in the magnetic field so that a new *C* = +1 edge state can be involved in transport. The Landau levels guarantee the formation of Chern band gaps inside the conduction or valence bands, whereas the Zeeman effect ensures the inversion of the original trivial bands in a magnetic field. Only in this case the universal tendency towards a zero Hall plateau state throughout the Chern insulator and the QH regimes can be well explained.

Naively, a zero Hall resistance in magnetic fields can be also attributed to other origins such as the coexistence of electron-hole puddles in a magnetic field. But a careful analysis of the transport data can rule it out. First, the zero Hall resistance due to the exact cancellation between electron and hole puddles is an accidental state that cannot form a broad *R*_*yx*_ = 0 plateau in a wide range of magnetic fields and *V*_*g*_. It cannot explain the convergence of *R*_*xx*_ towards the 0.5 *h*/*e*^2^ plateau in the initial stage of zero Hall plateau state either. In addition, a trivial insulator is not a likely explanation either because it will exhibit diverging *R*_*yx*_ in the strong magnetic field in dc-transport measurement, rather than a broad zero Hall plateau^45,46^. The presence of edge transport throughout the magnetic field regime, as well as the low-temperature saturation of *R*_*xx*_ for the different magnetic fields, also distinguish the zero Hall plateau state from a trivial insulator, as shown in Supplementary Fig. 2b. Notably, it is plausible that a spin-polarized edge mode with ballistic transport length beyond our longest channel length ~10.8 μm emerges in a high magnetic field. It may also lead to a plateau-like feature with quantized *R*_*xx*_. Further studies are required to completely rule out this possibility.

In conclusion, we realize a zero Hall plateau state in the MnBi_2_Te_4_ Chern insulator state when a strong magnetic field is applied. The robust zero Hall plateau against magnetic field and *V*_*g*_, as well as the nonlocal transport measurements, suggest this state is composed of two counter-propagating edge states that arise from the emergence of a new Chern bandgap. The zero Hall plateau state discovered in the magnetic field in MnBi_2_Te_4_ represents a unique quantum transport phenomenon generated by the intricate interplay among intrinsic magnetism, external magnetic field, and nontrivial band topology.

## Methods

### Crystal growth

High-quality MnBi_2_Te_4_ single crystals were grown by a direct mixture of Bi_2_Te_3_ and MnTe with the ratio of 1:1 in a vacuum-sealed silica ampoule. After first heated to 973 K, the mixture is slowly cooled down to 864 K, followed by a long period of the annealing process. The phase and crystal quality are examined by X-ray diffraction on a PANalytical Empyrean diffractometer with Cu Kα radiation.

### Device fabrication

MnBi_2_Te_4_ flakes were mechanically exfoliated onto 285 nm-thick SiO_2_/Si substrates by using the Scotch tape method. Before exfoliation, all SiO_2_/Si substrates were pre-cleaned in air plasma for 5 min with ~125 Pa pressure. Thick flakes around the target sample area were manually scratched off by using a sharp needle. A 270 nm thick Poly(methyl methacrylate) (PMMA) layer was spin-coated on the exfoliated film before electron-beam lithography (EBL). After the EBL, 53 nm thick metal electrodes (Cr/Au, 3/50 nm) were deposited using a thermal evaporator connected to an argon-filled glove box with the O_2_ and H_2_O levels lower than 0.1 PPM. Throughout the fabrication and sample transfer process, the device was covered by PMMA to avoid direct contact with air. Four devices with 7-SL and 6-SL MnBi_2_Te_4_ denoted as device #7-SL-1, #7-SL-2, #7-SL-3 and #6-SL-1 were measured in a pulsed magnetic field. The data shown in the main figures are taken from device #7-SL-1, and that of other devices are documented in Supplementary Fig. 1 to Fig. 11.

### Transport measurement

High-field electrical transport measurements were performed in a ^4^He cryostat with a base temperature of 2 K in Wuhan National High Magnetic Field Center. A pulsed DC current of 4 μA was generated by a Yokogawa GS610 current source. An uncertainty of 250 Ω arising from the high rate of field sweep (1000 T/s) in pulsed magnetic field measurements and imperfect cancellation of measurement circuit was estimated according to the real geometry of the circuit. The absence of hysteresis in low-field transport data of the 7-SL MnBi_2_Te_4_ samples is due to the fast field sweeping rate. Low-field calibration of the 6-SL thick device was performed in a commercial ^4^He cryostat with a superconducting magnet up to 9 T. The longitudinal and Hall voltages were measured simultaneously by using lock-in amplifiers with an AC current of 200 nA generated by a Keithley 6221 current source. The back gate was applied by a Keithley 2400 source meter. To eliminate the effect of electrode misalignment, the measured four-terminal longitudinal and transverse resistances were symmetrized and antisymmetrized with respect to the magnetic field.

### Theoretical calculation

First-principles calculations were performed in the framework of density functional theory (DFT) using the Vienna ab initio Simulation Package^47^. The plane-wave basis with an energy cutoff of 350 eV was adopted, in combination with the projected augmented wave (PAW) method. The Monkhorst-Pack ***k***-point mesh of 9 × 9 × 3 was adopted in the self-consistent calculation with the inclusion of spin-orbit coupling. The modified Becke-Johnson (mBJ) functional^48^ was employed to improve the description of the electronic band structure in the ferromagnetic (FM) MnBi_2_Te_4_ bulk. The DFT-D3 method^49^ was used to describe van der Waals (vdW) interactions between neighboring septuple layers in MnBi_2_Te_4_. The tight-binding models derived from the FM bulk were used to model thin films. Maximally localized Wannier functions were constructed from first-principles calculations of the FM bulk, and the tight-binding Hamiltonian of the FM bulk was obtained. Then, tight-binding Hamiltonians of thin films were constructed by cutting slabs from the bulk. Band structures, topological properties, edge state calculations^50^, and effective ***k***·***p*** Hamiltonians of MnBi_2_Te_4_ thin films were computed based on the tight-binding Hamiltonians.

## Supplementary information

Supplementary Information

## Data Availability

All raw and derived data used to support the findings of this work are available from the authors on request.
