# Supplementary material for: Magnetic-field-induced robust zero Hall plateau state in MnBi2Te4 Chern insulator
Source: Nat Commun. 2021 Jul 30;12:4647. doi: 10.1038/s41467-021-25002-x (PMC8324822; doi:10.1038/s41467-021-25002-x)
Supplement: Supplementary file 1 — Supplementary Information [file 41467_2021_25002_MOESM1_ESM.pdf]

Supplementary Information

Magnetic-field-induced robust zero Hall plateau state in $MnBi_2Te_4$ Chern insulator

Chang Liu[1,2†], Yongchao Wang[3,†], Ming Yang[4†], Jiahao Mao[1], Hao Li[5,6], Yaoxin Li[1], Jiaheng Li[1], Haipeng Zhu[4], Junfeng Wang[4], Liang Li[4], Yang Wu[6,7], Yong Xu[1,8,9*], Jinsong Zhang[1,9*], Yayu Wang[1,9*]

[1]*State Key Laboratory of Low Dimensional Quantum Physics, Department of Physics, Tsinghua University, Beijing 100084, P. R. China*

[2]*Beijing Academy of Quantum Information Sciences, Beijing 100193, P. R. China*

[3]*Beijing Innovation Center for Future Chips, Tsinghua University, Beijing 100084, P. R. China*

[4]*Wuhan National Magnetic Field Center, Huazhong University of Science and Technology, Wuhan 430074, P. R. China*

[5]*School of Materials Science and Engineering, Tsinghua University, Beijing, 100084, P. R. China*

[6]*Tsinghua-Foxconn Nanotechnology Research Center, Department of Physics, Tsinghua University, Beijing 100084, P. R. China*

[7]*Department of Mechanical Engineering, Tsinghua University, Beijing 100084, P. R. China*

[8]*RIKEN Center for Emergent Matter Science, Wako, Saitama 351-0198, Japan*

[9]*Collaborative Innovation Center of Quantum Matter, Beijing, P. R. China*

[†] These authors contributed equally to this work.

* Emails: yongxu@tsinghua.edu.cn; jinsongzhang@tsinghua.edu.cn; yayuwang@tsinghua.edu.cn

Supplementary Note:

1. Low-field transport properties of Device #7-SL-1

2. Temperature dependent transport behavior for Device #7-SL-1

3. Pulsed field transport data for Device #7-SL-2

4. Basic low-field transport calibrations for Device #6-SL-1

5. V_g dependent R_{xx} and R_{yx} for Device #6-SL-1 in pulsed magnetic fields

6. Experimental phase diagram of the 6-SL device

7. Schematic band structure evolution for different situations of Zeeman effect

8. Effective Hamiltonian and calculated Landau levels

9. Reproducible nonlocal transport data for Device #7-SL-3

Supplementary Note 1:

Low-field transport properties of Device #7-SL-1

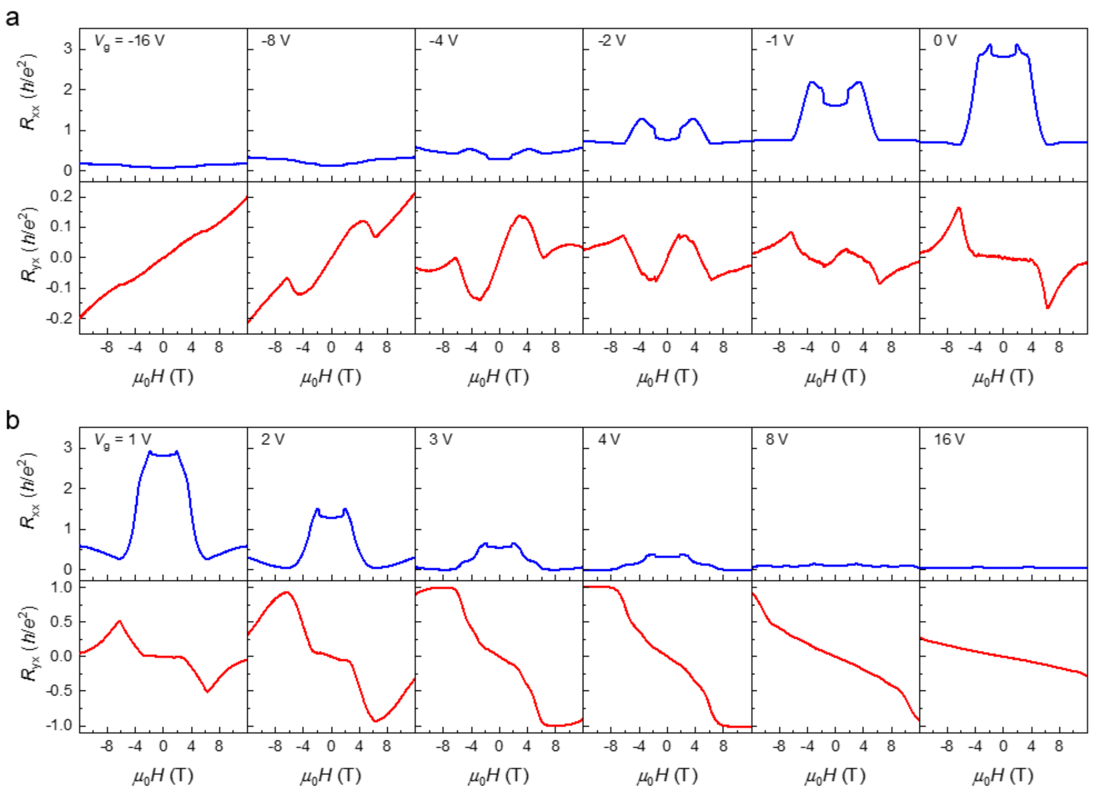

Supplementary Fig. 1 | Low-field transport properties of the 7-SL $MnBi_2Te_4$ device S1. Magnetic field dependent R_{xx} and R_{yx} measured at 2 K for -16 V ≤ V_g ≤ 0 V (a), and 1 V ≤ V_g ≤ 16 V (b).

In this work, we investigated the transport properties of four $MnBi_2Te_4$ samples with different thickness and sample size, including three 7-SL devices (#7-SL-1, #7-SL-2 and #7-SL-3) and one 6-SL device (#6-SL-1). The results presented in the main figures were taken from Device #7-SL-1. Due to the limited space, only part of the data are displayed.

In this session, we present the complete data set of the magnetic field dependent R_{xx} and R_{yx} at varied V_gs, as shown in Supplementary Figs. 1a and 1b, respectively. In the regime of $V_g \leq 0$ V, the low-field R_{xx} increases with V_g, and the low-field slope of R_{yx} changes sign at $V_g = 0$ V. With the increase of magnetic field, a series of jumps appear in both R_{xx} and R_{yx}, corresponding to the sequential flipping events of magnetic

moments in each individual SL of $MnBi_2Te_4$. Both R_{xx} and R_{yx} show abrupt changes at magnetic field of 6 T, indicating the dramatic change of band structure upon entering the FM state. At $V_g = 4$ V, the system enters the Chern insulator phase when FM order forms, as discussed in the main text. Further increase of V_g raises the position of E_F towards the conduction band, leading to more conductive properties. In addition to the decrease of R_{xx}, $|R_{yx}|$ also deviates from the quantized plateau (h/e^2) and develops a negative slope that is characteristic of ordinary Hall effect in a 2D electron gas.

Supplementary Note 2:

Temperature dependent transport behavior for Device #7-SL-1

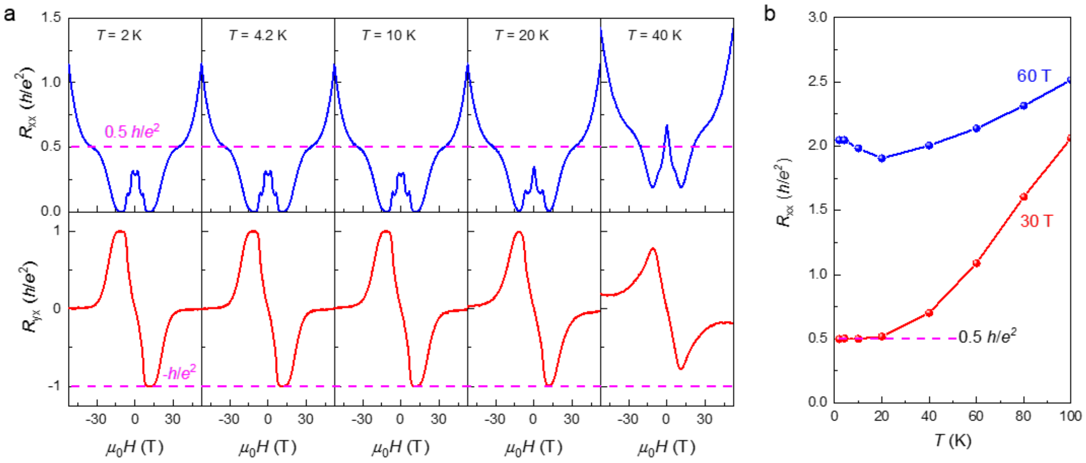

Supplementary Fig. 2 | Temperature dependent transport behavior for Device #7-SL-1 at V_g = 4 V. a, Magnetic field dependent R_{xx} and R_{yx} measured at 2 K. **b**, Temperature dependent R_{xx} in different magnetic fields.

Supplementary Fig. 2a shows the magnetic field dependent R_{xx} and R_{yx} for Device #7-SL-1 at $V_g = 4$ V and varied temperatures from $T = 2$ K to 40 K. Both the $C = -1$ and $C = 0$ phases are highly robust against temperature. Even at $T = 20$ K, the quantization of R_{yx} reaches as high as -0.991 h/e^2 for the $C = -1$ phase. The zero Hall plateau for the helical $C = 0$ phase and the quantization of R_{xx} at 0.5 h/e^2 near 30 T are also very clear. As the temperature is increased further, significant changes appear in R_{xx} at low magnetic field regime, which is related to the weakening of AFM order above the Neel temperature $T_N \sim 25$ K. Meanwhile, both the features of the $C = -1$ and $C = 0$ phases

are weakened. These results unambiguously show the robustness of the Chern insulator phase in high magnetic field. In Supplementary Fig. 2b, we display the temperature dependent R_{xx} for the $C = 0$ phase in different magnetic fields. At the onset field of about 30 T, the scattering between the helical edge states is weak, so the half-quantized R_{xx} persists in a broad temperature regime of 20 K, as marked by the magenta dashed line. At 60 T where the scattering leads to more insulating behavior, the value of R_{xx} is much larger. However, fundamentally different from trivial insulator with diverging R_{xx} with lowering temperature, R_{xx} increases slightly and saturates at the ground state. The temperature dependent R_{xx} clearly shows that the helical $C = 0$ phase in our work is not a trivial insulator.

Supplementary Note 3:
Pulsed field transport data for Device #7-SL-2

To further demonstrate the reproducibility of the main results presented in the main text, we measured another 7-SL device (#7-SL-2) in pulsed magnetic fields. The optical image of this device is shown in Supplementary Fig. 3a. During the cooling process, one current lead had bad connection to electrode 1 in the image, therefore the current was applied from electrode 6 to 4. The measurement setup for R_{xx} and R_{yx} are marked in the figure. Supplementary Fig. 3b displays the magnetic field dependent R_{xx} and R_{yx} measured at different V_gs. The overall behaviors of both R_{xx} and R_{yx} are consistent to that observed in Device #7-SL-1 (see Fig. 2 in the main figures for details), although there are some quantitative differences in the detailed magnetic field and gate voltage dependences.

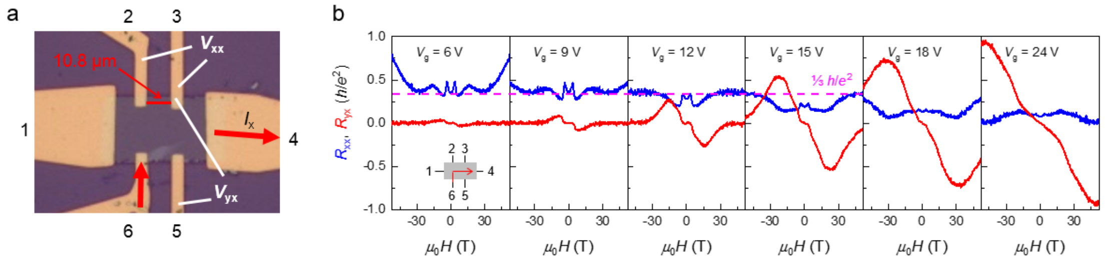

Supplementary Fig. 3 | Magnetic field dependent R_{xx} and R_{yx} measured in pulsed

fields for Device #7-SL-2 at various V_gs. a, Optical images and measurement setups for Device #7-SL-2. **b**, Magnetic field dependent R_{xx} and R_{yx} for different V_gs. The magenta dashed lines mark the quantized values of ⅓ h/e^2 expected for R_{xx} at the onset of zero Hall plateau.

The most striking observation is that a broad zero Hall plateau also forms in the high field regime in Device #7-SL-2. With the increase of V_g, the zero Hall plateau becomes narrower, and the C = -1 plateau starts to emerge. Notably, a broad quantized R_{xx} ~ ⅓ h/e^2 plateau appears at the onset magnetic field of the C = 0 phase, as marked by the magenta broken line. This is exactly the quantized resistance expected for helical edge states in such electrode configuration.

Another important point is that Device #7-SL-2 has larger size than Device #7-SL-1 shown in the main text. The distance between the two R_{xx} electrodes is 10.8 μm, whereas for Device #7-SL-1 it is only 6.7 μm. The quantization of R_{xx} at the onset of the zero Hall plateau in samples with larger size is another strong evidence to support the helical nature of the C = 0 phase.

Supplementary Note 4:
Basic low-field transport calibrations for Device #6-SL-1

In this session, we present the magnetic field dependent transport results measured at different temperatures and V_gs for an even-number-layer sample (Device #6-SL-1). The variation of film thickness mainly affects the low-field properties when $MnBi_2Te_4$ is in the AFM state, which is not the focus of this work. In the FM state, both 6-SL and 7-SL samples are expected to exhibit the Chern insulator behavior with robust quantized Hall plateau[1,2].

Supplementary Fig. 4 shows the magnetic field dependent R_{xx} and R_{yx} for the 6-SL device at varied V_gs. A unique feature for 6-SL $MnBi_2Te_4$ device is that the axion insulator phase is expected at low-field regime when E_F is tuned to the CNP, as demonstrated in our previous report[1]. The overall behaviors of this device is similar to

that of the three 7-SL devices. Both R_{xx} and R_{yx} show systematic evolutions in response to magnetic field. At the magnetic-field-driven AFM to FM transition, R_{xx} exhibits a dramatic decrease, accompanied by an abrupt jump in R_{yx}. At $V_g < 36$ V, the low-field R_{xx} increases with the increase of V_g, and R_{yx} exhibits linear behavior with overall positive slope in magnetic field. At 42 V $\leq V_g \leq$ 46 V, the system enters the regime of axion and Chern insulator phases. In the low-field regime for the axion insulator phase, the value of R_{xx} reaches as high as 5 h/e^2, followed by a sharp decrease to zero as magnetic field exceeds 6 T. Accordingly, R_{yx} exhibits a sharp transition from the zero plateau to the $R_{yx} = -h/e^2$ plateau. As V_g is further increased, the system deviates from the axion and Chern insulator phases, as a natural result of the increased contributions from electron-type charge carriers. At $V_g = 59$ V, R_{xx} is reduced to as low as 1 h/e^2, and R_{yx} exhibits overall negative slope in both the low- and high-field regime. Noting that larger V_g is required in the 6-SL device for observing the Chern insulator phase, which suggests that the 6-SL device is much more hole-doped than the 7-SL device. This is because fabrication process tends to introduce holes in the pristine electron-doped bulk crystal, as is shown in our previous report[1].

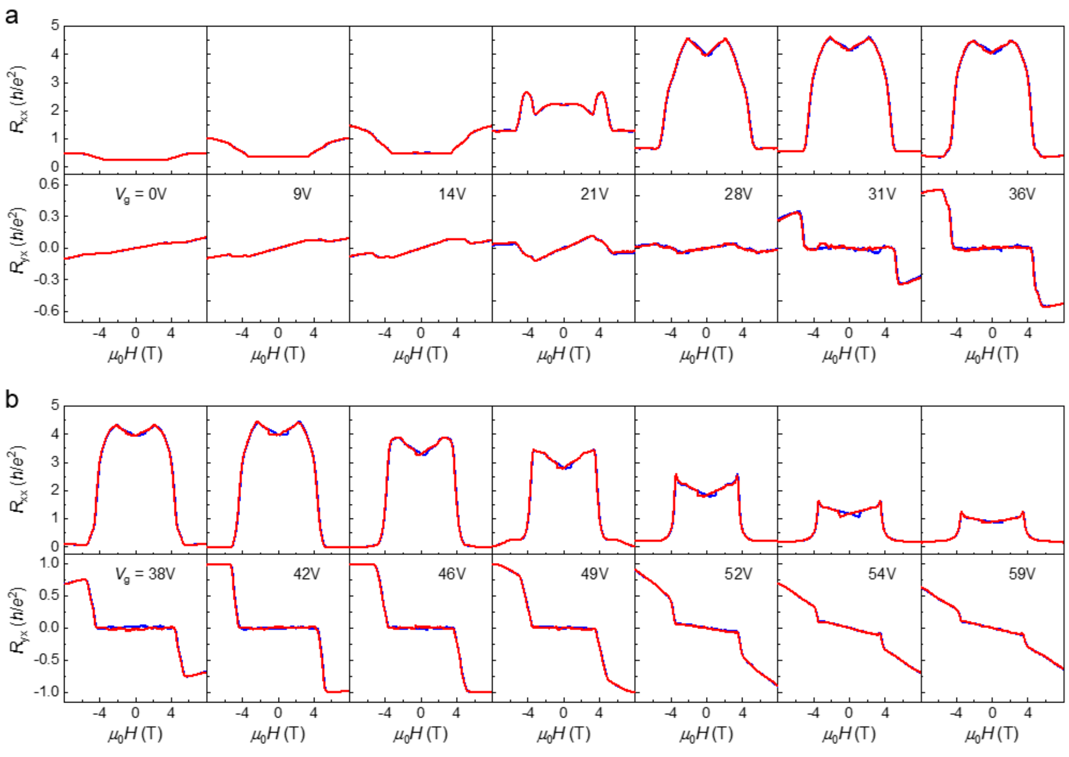

Supplementary Fig. 4 | Magnetic field dependent R_{xx} and R_{yx} at 1.6 K for varied

V_gs for Device #6-SL-1. Magnetic field dependent R_{xx} and R_{yx} measured at 2 K for 0 V ≤ V_g ≤ 36 V **(a),** and 38 V ≤ V_g ≤ 59 V **(b)**. The best regime for the Chern insulator phase lies in V_g from 42 V to 46 V.

Supplementary Fig. 5 displays the magnetic field dependent R_{xx} and R_{yx} at varied temperatures measured at V_g = 46 V for Device #6-SL-1. Slightly quantitative difference between the data shown here and that in Supplementary Fig. 4 is because this series of data was acquired after the V_g dependent measurements. Supplementary Fig. 5 clearly shows the zero-field R_{xx} increases with the decrease of temperature, reaching as high as 4 h/e^2 at the lowest temperature 1.6 K. Accompanied by the insulating behavior of R_{xx}, R_{yx} displays a wide zero Hall plateau at the same field range. Both behaviors are characteristics of the axion insulator phase. With the increase of magnetic field, the AFM state is driven to the FM state, and R_{xx} quickly drops to nearly zero. Accordingly, R_{yx} undergoes a sharp transition from the zero plateau to the R_{yx} = -h/e^2 plateau. The quantized R_{yx} and vanished R_{xx} undoubtedly demonstrate that the 6-SL $MnBi_2Te_4$ device is in the Chern insulator phase for the FM state. Remarkably, the quantization of the Chern insulator phase here is highly stable against the thermal activation. Even at 8 K, |R_{yx}| is still quantized at 0.997 h/e^2 and R_{xx} is as low as 0.009 h/e^2.

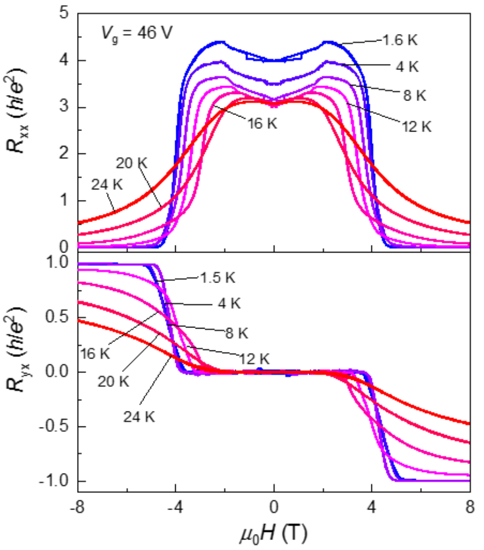

Supplementary Fig. 5 | Magnetic field dependent R_{xx} and R_{yx} at V_g = 46 V for varied

temperatures for Device #6-SL-4. At $\mu_0 H$ = 8 T, the Chern insulator phase persists to temperature as high as 8 K, with $|R_{yx}|$ higher than 0.997 h/e^2, and R_{xx} as small as 0.009 h/e^2.

Supplementary Note 5:
V_g dependent R_{xx} and R_{yx} for Device #6-SL-1 in pulsed magnetic fields

Supplementary Figs. 6a and 6b display the V_g dependent R_{xx} and R_{yx} in pulsed magnetic fields for the 6-SL device. The entire data set were acquired after the measurement at Tsinghua. As mentioned above, the electrical contact is fragile against the sample transfer process, in particularly after undergoing frequent thermal cycling process. One current contact is completely broken when transferred to the pulsed magnetic field facility. Thus, it is replaced by a side voltage contact during the pulsed field measurements. The layout of contact arrangement is displayed in the inset of Supplementary Fig. 6a. The current flows from contact 2 to contact 4. R_{xx} and R_{yx} are obtained simultaneously by measuring V_{65} and V_{35}. Such setup only quantitatively affects the value of R_{xx}, but would not affect the qualitative transport behaviors.

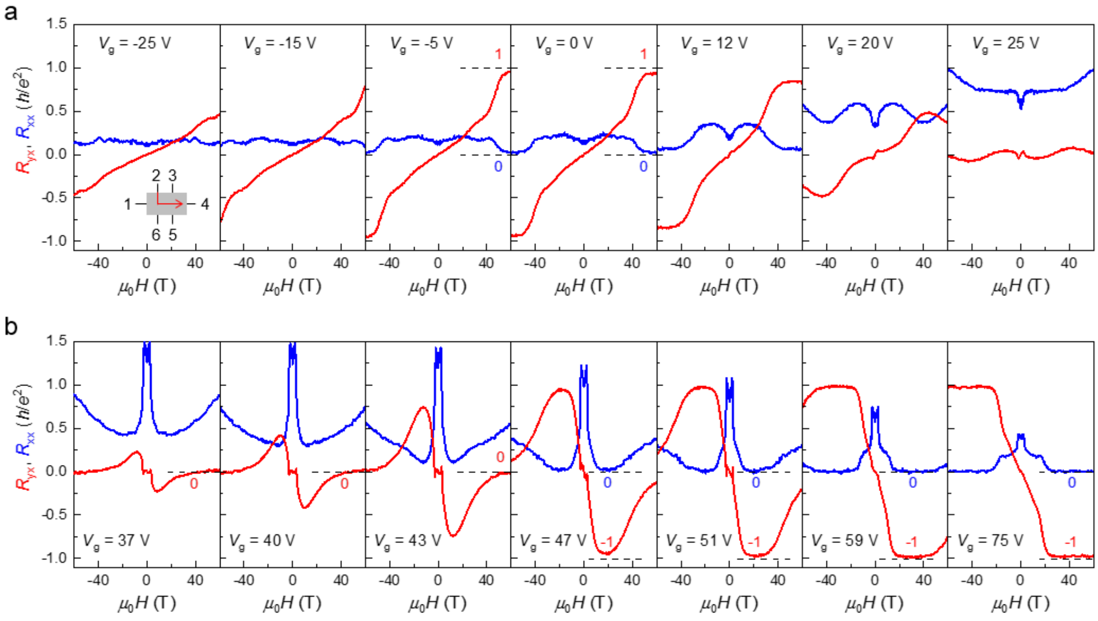

Supplementary Fig. 6 | The V_g dependent transport properties at 2 K for Device #6-SL-1 in pulsed magnetic fields. Magnetic field dependent R_{xx} (blue) and R_{yx} (red) measured at -25 V ≤ V_g ≤ 25 V **(a)** and 37 V ≤ V_g ≤ 75 V **(b)**. The C = +1 phase is most

pronounced for -5 V ≤ V_g ≤ 0 V, with R_{yx} = 0.94 ± 0.01 h/e^2 and R_{xx} = 0.03 ± 0.02 h/e^2 at 60 T. Starting from V_g = 47 V, the C = -1 phase shows up when magnetic field exceeds 20 T, with R_{yx} = -0.94 ± 0.01 h/e^2 and R_{xx} = 0.02 ± 0.02 h/e^2 at 60 T. The best quantization is realized at V_g ≥ 59 V, in which R_{yx} = -0.98 ± 0.01 h/e^2 and R_{xx} = 0 ± 0.02 h/e^2. The error bars are estimated from the amplitudes of the fluctuations in the signals.

Supplementary Fig. 6a shows the magnetic field dependent R_{xx} (blue) and R_{yx} (red) measured at V_g ranging from -25 V ≤ V_g ≤ 25 V. In this V_g range, the transport is mainly conducted by hole-like carriers, as reflected by the overall positive slope of R_{yx}. Starting from V_g = -5 V, pronounced QH state with C = +1 appears in high magnetic fields. At 60 T, R_{yx} reaches 0.94 ± 0.01 h/e^2, and R_{xx} drops to as low as 0.03 ± 0.02 h/e^2. Such feature is also observed in the 7-SL thick devices, as presented in Fig. 2 in the main figures. As V_g is further increased to 37 V, the C = 0 phase appears.

Although Device #6-SL-1 exhibits qualitatively consistent behaviors as that of the three 7-SL devices, there are several quantitative differences. Firstly, in the 6-SL device, 60 T is not large enough for the simultaneous observation of C = -1 and C = 0 phases. The plateau width of the C = 0 phase is much narrower than that of the 7-SL devices, and only appears in the highest magnetic field regime. Secondly, unlike the behaviors for the 7-SL devices (Fig. 2 in the main figures and Supplementary Fig. 2), where the C = 0 phase becomes broader and the C = -1 phase shifts towards the low-field side as holes are injected, the C = 0 phase for Device #6-SL-1 remains in a narrow high field regime. Several possible reasons could account for these discrepancies. One intrinsic reason is that the gap size of the trivial quantum well bands in the 6-SL sample is larger than that of the 7-SL sample, thus a much larger magnetic field is required to completely suppress the C = -1 phase. A more likely reason is that the 6-SL device was doped due the aging effect. The unavoidable exposure to air during the sample transfer process may introduce chemical doping to the sample. The enlarged V_g and magnetic field for observing the C = -1 phase in pulsed field measurements provide several informative evidences. The required V_g and magnetic field are 42 V and 5 T for the measurement in static magnetic fields (Supplementary Fig. 4), whereas over 47 V and 20 T are required

for the pulsed magnetic fields (Supplementary Fig. 6). Moreover, the level of quantization also decreases from more than 0.99 h/e^2 at V_g = 42 V (Supplementary Fig. 4) in the static measurements to 0.94 h/e^2 at V_g = 47 V (Supplementary Fig. 6) in the pulsed measurements, which undoubtedly indicates that the sample quality has indeed seriously degraded.

To avoid the influence of the degradation of sample quality on our main conclusion, we performed all the measurements on Device #7-SL-1 in pulsed magnetic fields. We first characterize the detailed gate voltage dependence by applying a pulse field to 12 T, and then extend the measurements up to 61.5 T for the representative gate voltages in the interesting regimes. The results presented in the main text and figures are all from the Device #7-SL-1.

Supplementary Note 6:
Experimental phase diagram of the 6-SL device

Supplementary Fig. 7 displays the experimental phase diagram for Device #6-SL-1 summarized from the values of R_{yx}, dR_{yx}/dH and R_{xx}. Starting from V_g = -30 V, hole-type carriers are gradually depleted with increasing V_g. At V_g = -5 V, QH phase with C = 1 appears in the high-field regime, as represented by red and white in Supplementary Figs. 7a and 7b. As V_g is increased to about 30 V, the C = 0 phase appears and persists to 60 T. Similar to the phase diagram of 7-SL devices, the C = 0 phase occupies the largest portion of the phase diagram and is highly stable with respect to the changes of both V_g and magnetic field. Further increase of V_g leads to the appearance of the C = -1 and C = -2 phase. Compared to the C = 1 phase in the hole-type regime, the area of C = -1 phase is much wider. The 6-SL and 7-SL devices manifest consistent phase diagrams, indicating that the observed phenomena are universal for $MnBi_2Te_4$ in the 2D limit.

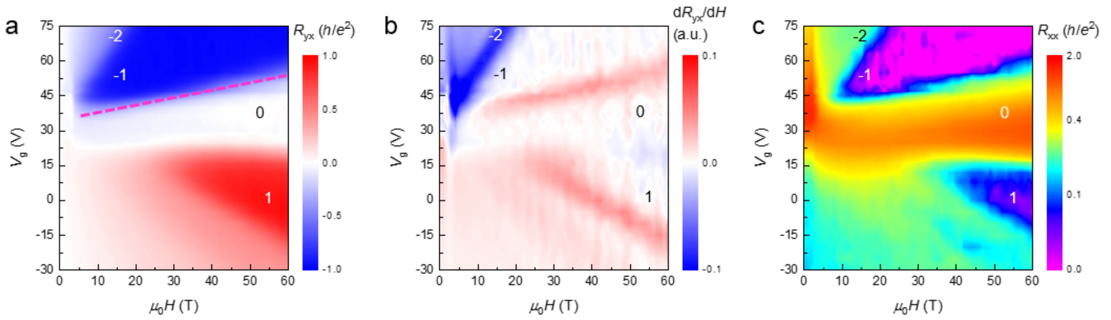

Supplementary Fig. 7 | Experimental phase diagram for Device #6-SL-1. The measured R_{yx} (**a**), dR_{yx}/dH (**b**), and R_{xx} (**c**) values as functions of magnetic field and V_g.

Supplementary Note 7:
Schematic band structure evolution for different situations of Zeeman effect

In Supplementary Fig. 8, we compare the band structure evolution in magnetic field in the three situations of without Zeeman effect, with small and with large Zeeman effect. Without Zeeman effect, the energy gap is only determined by the cyclotron motion of electrons, which increases in magnetic field. The gap size enlarges as the formation of Landau levels, which is also demonstrated by the calculated Landau level spectrums. As shown in Supplementary Fig. 8a, for the C = -1 phase with E_F lying in the band gap (green shadow regime), it will persists in magnetic field. And for E_F lying in the valence band, increasing magnetic field will further stabilizes the C = -1 phase. Obviously, both are absent in our experiment. Supplementary Fig. 8b shows the case when there is a small Zeeman effect. In this case, the two blue bands move oppositely in magnetic field and the band gap progressively decreases. It is clear that for E_F lying in the valence band (V_g = 0 V in our experiment), with the formation of n = 0 Landau levels, a C = 0 phase with a broad zero Hall plateau forms in magnetic field, as observed in our experiment. However, because the band gap does not close, the C = -1 Chern insulator phase for E_F lying in the gap is unaffected in magnetic field (green shadow regime). As long as there is no band inversion, such V_g regime for C = -1 phase is persistent forever. Therefore, considering Landau levels and a small Zeeman effect is also insufficient for our experiment. To fully explain our experimental data, not only Zeeman effect is indispensable, but also its magnitude is required to be sufficiently

large so that a band inversion can be induced, as shown in Supplementary Fig. 8c. In this case, all the experimental observation in our work can be well explained.

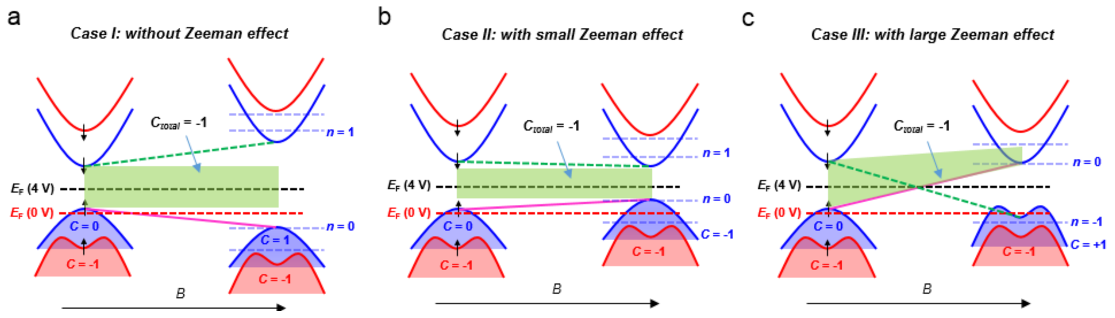

Supplementary Fig. 8 | Schematic band structure evolution without Zeeman effect (a), with small Zeeman effect (b) and with sufficient large Zeeman effect (c). The green shadow regime represents the V_g range where the C = -1 Chern insulator phase persists in strong magnetic field.

Supplementary Note 8:
Effective Hamiltonian and calculated Landau levels

To understand the experimental findings, we performed first-principles electronic structure calculations and studied the magnetic/topological properties by the effective Hamiltonian method. In zero magnetic field, the bulk of $MnBi_2Te_4$ is an AFM TI, and the (111) films have topological surface states gapped by magnetism. An interesting property of $MnBi_2Te_4$ in AFM state is that the electronic coupling between neighboring SLs is largely suppressed by the parity-time (*PT*) symmetry[3]. By applying strong out-of-plane magnetic fields, an AFM to FM phase transition happens, which results in *PT* symmetry breaking and thus significantly enhances the interlayer coupling. This could drive a topological phase transition from AFM TI to Weyl semimetal, according to theoretical calculations[4,5]. Theoretically, the low-energy physics of FM $MnBi_2Te_4$ films are described by a four-band model based on the four quantum well states near the E_F. Figure 3c in the main text displays the theoretical band structure for the 7-SL $MnBi_2Te_4$ film with FM order. Remarkably, the second highest valence band displays an obvious M-shape feature near Γ, which is a signature of topological band inversion. Topological

edge-state calculations reveal that the band inversion indeed happens between this valence band and a conduction band, as shown in Supplementary Fig. 9. We also computed the topological invariant and got C = -1, in good agreement with the experiment. Bands near the Fermi level are mainly contributed by the p_z orbitals of Te atoms, which have small orbital angular momentum. For out-of-plane polarized Mn^{2+} moments, the z components of spin angular momentum s_z of the first/second conduction band minimum and valence band maximum (named CBM_1/CBM_2 and VBM_1/VBM_2) are negative and positive, respectively, corresponding to total angular momentum J_z = -1/2 and +1/2 (Fig. 3d in main text) as found by first-principles calculations.

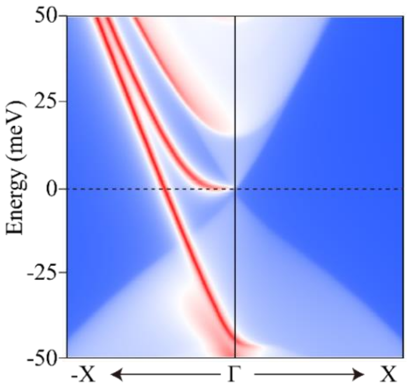

Supplementary Fig. 9 | Edge states along (100) direction in the 7-SL FM $MnBi_2Te_4$ film.

Using the bases of $|\mathrm{CBM}_1\rangle$, $|\mathrm{VBM}_1\rangle$, $|\mathrm{VBM}_2\rangle$, and $|\mathrm{CBM}_2\rangle$, which have J_z = −1/2, +1/2, +1/2 and −1/2, respectively, we constructed a low-energy effective Hamiltonian by the ***k·p*** approach[6]:

$$H(k) = \begin{bmatrix} h_+(k) & 0 \\ 0 & h_-(k) \end{bmatrix},$$

where $h_\pm(k) = \epsilon_\pm(k)\sigma_0 + \boldsymbol{d}_\pm \cdot \boldsymbol{\sigma}$, $\sigma_{x,y,z}$ is the Pauli matrix, $\epsilon_\pm(k) = \epsilon_0^\pm + D_\pm k^2$, $k^2 = k_x^2 + k_y^2$, $\boldsymbol{d}_\pm = (A_\pm k_x, \pm A_\pm k_y, M_0^\pm - M_2^\pm k^2)$, and $M_2^\pm < 0$ is assumed. Importantly, $h_\pm(k)$ gives Chern number $C_\pm = 0$ when $M_0^\pm > 0$ and $C_\pm = \pm 1$ when $M_0^\pm < 0$ (ref. [7,8]). By fitting bands from *ab initio* calculations, we obtained a positive M_0^+ and a negative M_0^-, giving $C_+ = 0$ and $C_- = -1$. The total Chern number is $C = C_+ + C_- = -1$. As the two bands given by $h_-(k)$ are relatively far away from the E_F, magnetic

responses of the system are mainly determined by $h_+(k)$. The fitting parameters of $h_+(k)$ are $\epsilon_0^+ = 0$ eV, $D_+ = 16.0\ \mathrm{eV}\cdot\text{Å}^2$, $A_+ = 1.5\ \mathrm{eV}\cdot\text{Å}$, $M_0^+ = 1.5\times10^{-3}$ eV, and $M_2^+ = -16.8\ \mathrm{eV}\cdot\text{Å}^2$.

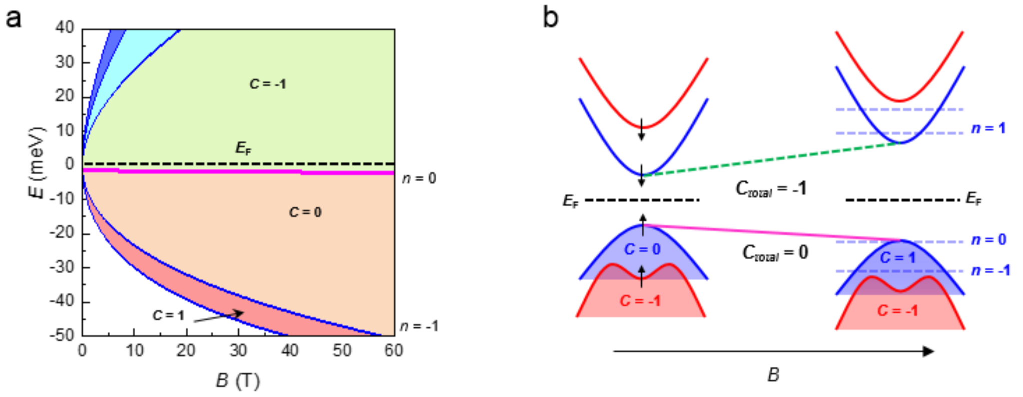

Supplementary Fig. 10 | Calculated Landau level spectrums and Chern numbers in magnetic field without Zeeman effect (a) and schematic band structure evolution (b).

Then we studied the influence of external magnetic field $\boldsymbol{B} = (0, 0, B)$ based on $h_+(k)$. We chose the Landau gauge $\boldsymbol{A} = (-By, 0, 0)$ and included the orbital effect by replacing the electron momentum $\boldsymbol{p} \to \boldsymbol{p} + e\boldsymbol{A}$, where e is the elementary charge ($e > 0$). The Zeeman effect is described by $H_{\mathrm{Zeeman}} = -\frac{1}{2} g\mu_B B\sigma_z$, where g is the g-factor ($g \approx 2$ for free electrons) and μ_B is the Bohr magneton. The energy spectra of Landau levels for $h_+(k)$ can be analytically calculated[9]:

$$E_n = \begin{cases} \epsilon_0^+ + \frac{eB}{\hbar}(2nD_+ + M_2^+) \pm \sqrt{2n\frac{eB}{\hbar}A_+^2 + \left(M_0^+ - \frac{1}{2}g\mu_B B - \frac{eB}{\hbar}(D_+ + 2nM_2^+)\right)^2}, \\ n = 1, 2, 3\ldots \\ \epsilon_0^+ - M_0^+ + \frac{1}{2}g\mu_B B + \frac{eB}{\hbar}(D_+ + M_2^+),\ \ n = 0. \end{cases}$$

If neglecting the Zeeman effect by selecting $g = 0$, one would always get the $C = -1$ phase ($C_+ = 0$ and $C_- = -1$) at the charge neutral regime, as displayed in Supplementary Fig. 10a, even in a large magnetic field, which obviously contradicts with the experiment. Supplementary Fig. 10b shows the schematic illustration of the band structure evolution in magnetic field without Zeeman effect. It clearly shows that

as long as the E_F (black dashed line) is in the band gap (corresponding to the V_g range in Fig. 2a), it will remain forever in the C = -1 phase in high magnetic field. It is thus concluded that the Zeeman-effect-induced band inversion plays a crucial role in driving the C = - to C = 0 phase transition. Notably, it is difficult to evaluate the exact g-factor by first-principles calculations. By fitting the theoretical phase diagram with experiment and referring to previous experimental results in conventional TIs[10-12], $g = 10$ is selected in our calculations, which is on the same order of magnitude as Bi_2Te_3 (ref. [13]).

Supplementary Note 9:
Reproducible nonlocal transport data for Device #7-SL-3

In Supplementary Fig. 11, we display the results of four-probe and two nonlocal measurements with different configurations in a third 7-SL device. As the increase of magnetic field, R_{yx} decreases from $-h/e^2$ towards 0, accompanied by the saturation of R_{xx} towards 0.5 h/e^2, highly consistent with the data of Device 7-SL-1 shown in Fig. 2 in the main figures. Noting that the transition from the C = -1 to C = 0 phase in this device is not as sharp as that in Device 7-SL-1. This broad transition accidently allows us to observe the signature of quantized R_{xx} in nonlocal measurements in a broader magnetic field range, due to the weak scattering between the counter-propagating edge states at the onset field regime. Supplementary Figs. 10b and 10c display the nonlocal measurements results using the same configurations as Fig. 4b in the main figures. Clearly, broad R_{xx} plateaus with different quantized values are observed. These results unambiguously demonstrate the reproducibility of the nonlocal data.

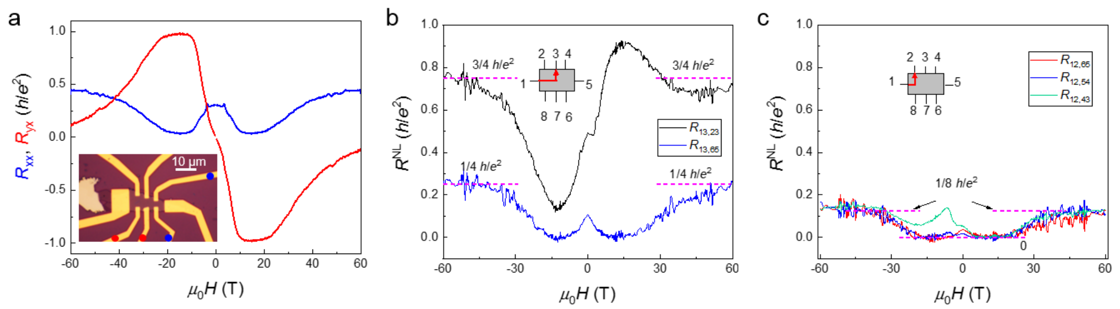

Supplementary Fig. 11 | Nonlocal transport data for Device #7-SL-3. a, Magnetic field dependence of R_{xx} and R_{yx} in four-probe measurement. The inset shows the optical image of this device. The blue and red dots denote the selected contacts for R_{yx} and R_{xx} measurements. **b,** Nonlocal measurements with current flowing between electrodes 1 and 3. Depending on the position of the voltage probes, the resistance is nearly quantized at 1/4 and 3/4 h/e^2. **c,** Nonlocal measurements with current flowing between electrodes 1 and 2. All the R_{xx} converge to 1/8 h/e^2. The expected quantized values of R_{xx} are denoted by the magenta broken lines.

References

1. Liu C., Wang Y., Li H., Wu Y., Li Y., Li J*., et al.* Robust axion insulator and Chern insulator phases in a two-dimensional antiferromagnetic topological insulator. *Nat Mater*. **19**, 522-527 (2020).

2. Deng Y., Yu Y., Shi M. Z., Guo Z., Xu Z., Wang J*., et al.* Quantum anomalous Hall effect in intrinsic magnetic topological insulator MnBi2Te4. *Science*. **367**, 895-900 (2020).

3. Li J. H., Wang C., Zhang Z. T., Gu B. L., Duan W. H., Xu Y. Magnetically controllable topological quantum phase transitions in the antiferromagnetic topological insulator $MnBi_2Te_4$. *Phys. Rev. B*. **100**, 121103(R) (2019).

4. Li J., Li Y., Du S., Wang Z., Gu B. L., Zhang S. C*., et al.* Intrinsic magnetic topological insulators in van der Waals layered $MnBi_2Te_4$-family materials. *Sci. Adv.* **5**, eaaw5685 (2019).

5. Zhang D., Shi M., Zhu T., Xing D., Zhang H., Wang J. Topological Axion States in the Magnetic Insulator $MnBi_2Te_4$ with the Quantized Magnetoelectric Effect. *Phys. Rev. Lett.* **122**, 206401 (2019).

6. Bernevig B. A., Hughes T. L., Zhang S. C. Quantum Spin Hall Effect and Topological Phase Transition in HgTe Quantum Wells. *Science*. **314**, 1757-1761 (2006).

7. Qi X. L., Wu Y. S., Zhang S. C. Topological quantization of the spin Hall effect in two-dimensional paramagnetic semiconductors. *Phys. Rev. B*. **74**, 085308 (2006).

8. Liu C. X., Qi X. L., Dai X., Fang Z., Zhang S. C. Quantum anomalous hall effect in $Hg_{1-y}Mn_yTe$ quantum wells. *Phys. Rev. Lett.* **101**, 146802 (2008).

9. Zhang S. B., Zhang Y. Y., Shen S. Q. Robustness of quantum spin Hall effect in an external magnetic field. *Phys. Rev. B*. **90**, 115305 (2014).

10. Analytis J. G., McDonald R. D., Riggs S. C., Chu J. H., Boebinger G. S., Fisher I. R.

Two-dimensional surface state in the quantum limit of a topological insulator. *Nat. Phys.* **6**, 960-964 (2010).

11. Taskin A. A., Ando Y. Berry phase of nonideal Dirac fermions in topological insulators. *Phys. Rev. B.* **84**, 035301 (2011).

12. Xiong J., Luo Y. K., Khoo Y. H., Jia S., Cava R. J., Ong N. P. High-field Shubnikov-de Haas oscillations in the topological insulator Bi_2Te_2Se. *Phys. Rev. B.* **86**, 045314 (2012).

13. Liu C. X., Qi X. L., Zhang H. J., Dai X., Fang Z., Zhang S. C. Model Hamiltonian for topological insulators. *Phys. Rev. B.* **82**, 045122 (2010).
